# LSD1 Promotes Prostate Cancer Cell Survival by Destabilizing FBXW7 at Post-Translational Level

**DOI:** 10.3389/fonc.2020.616185

**Published:** 2021-02-23

**Authors:** Xu-ke Qin, Yang Du, Xiu-heng Liu, Lei Wang

**Affiliations:** Department of Urology, Renmin Hospital of Wuhan University, Wuhan, China

**Keywords:** LSD1, FBXW7, prostate cancer, protein-protein interaction, tumor suppress

## Abstract

Prostate cancer (PCa) is the most common cancer in men and the fifth leading cause of cancer death worldwide. Unfortunately, castration-resistant prostate cancer (CRPCa) is incurable with surgical treat and prone to drug resistance. Therefore, it is of great importance to find a new target for treatment. LSD1 is up-regulated in PCa and related with prognosis. The high-expression LSD1 has been shown to be a potential target for treatment and is widely studied for its demethylase-activity. However, its demethylation-independent function remains to be elusive in PCa. Recent study shows that LSD1 can destabilize cancer suppressor protein FBXW7 without demethylation-function. Hence, we hope to investigate the impact of non-canonical function of LSD1 on PCa cell survival. We over-expressed FBXW7 gene through plasmid vector in LNCaP and PC3 cell lines and the result shows that up-regulated FBXW7 can suppress the viability of PC cell through suppressing oncoproteins, such as c-MYC, NOTCH-1. After FBXW7 function experiment on PC cell, we knock-down LSD1 gene in the same kinds of cell lines. In western blot assay, we detected that down-regulation of LSD1 will cause the increasing of FBXW7 protein level and decreasing of its targeting oncoproteins. And mRNA level of FBXW7 did not change significantly after LSD1 knock-down, which means LSD1 may destabilize FBXW7 by protein-protein interactions. Moreover, exogenous wild type LSD1 and catalytically deficient mutant K661A both can abrogate previous effect of LSD1 knock-down. Consequently, LSD1 may promote PC cell survival by destabilizing FBXW7 without its demethylase-activity. Next, we compared two kinds inhibitors, and found that SP-2509 (Allosteric inhibitor) treatment suppress the cancer cell survival by blocking the LSD1–FBXW7 interaction, which is an effect that GSK-2879552 (catalytic inhibitor) cannot achieve. This work revealed a pivotal function of LSD1 in PCa, and indicated a new direction of LSD1 inhibitor research for PCa treatment.

## Introduction

As of 2018, global cancer statistics show that prostate cancer has become the second most common cancer in men and the fifth leading cause of cancer death (1). In this disease, advanced prostate cancer is incurable and has become the second leading cause of cancer-related death in the United States (1). For localized prostate cancer, radical prostatectomy is always the first choice. However, androgen-deprivation therapy (ADT) is the standard treatment for patients with advanced prostate cancer. Unfortunately, the effect of ADT lasts for a limited time, and tumor cells will develop resistance to ADT treatment. Therefore, there is an urgent need to find new targets and treatment methods for the treatment of advanced prostate cancer (2, 3).

LSD1 is overexpressed in many human cancers and is associated with poor prognosis (4). Increasing studies indicate that LSD1 is a promising target of cancer therapy. Accordingly, a series of LSD1 inhibitors have been in clinical studies (5). So far, most of LSD1 studies focused on its demethylase activity and associated biological functions. Likewise, LSD1-based drug discovery focused on targeting LSD1’s demethylase activity (6). A recent study showed that LSD1 can regulate the stability of FBXW7 protein independent of its demethylation function (7). FBXW7 is an F-box protein and is responsible for substrate recognition in the SCF (Skp1-CUL1-F-box protein) E3 ubiquitin ligase complex. FBXW7 is a typical tumor suppressor and can target a series of key human oncoproteins, such as cyclin E, c-JUN, c-MYC, NOTCH-1, and MCL-1. It can cause ubiquitination of target genes and protease degradation (8). The study found that LSD1 is a pseudo substrate of FBXW7. After combination with LSD1, FBXW7 did not promote the degradation of LSD1. AND LSD1 did not change the function of FBXW7, but triggered the self-ubiquitination of FBXW7.

Prostate cancer is also a cancer that highly expresses LSD1. However, the interaction between LSD1 and FBXW7 has not been reported in prostate cancer. Our study investigated the expression of LSD1 and FBXW7 in PCa specimens, and correlated their expression levels with clinicopathological data in the patient cohort. At the same time, the LSD1 inhibition was used to observe the changes in FBXW7 protein levels. Our research has shown that LSD1 destabilizes FBXW7 in a way that does not depend on demethylase, thereby promoting the survival of CRPC cells.

## Materials and Methods

### Patients and Specimens

Fifty two pairs of prostate cancer tissues and adjacent normal tissues were obtained from patients undergoing urological surgery in Wuhan University People’s Hospital from 2015 to 2018. All research protocols were approved by the Ethics Committee of Renmin Hospital of Wuhan University. Sign informed consent before surgery and collect relevant clinical data of patients. All tissue samples were immediately frozen in liquid nitrogen and then stored at −80°C until Immunohistochemical analysis.

### Immunohistochemical Analysis and Evaluation

(IHC) using specific antibodies for LSD1 (mouse monoclonal, 1:50, Santa Cruz), FBXW7 (mouse monoclonal, 1:100, Cell Signal Technology). Serial sections (thickness 5 µm) were cut from the tissue blocks, deparaffinized in xylene, and hydrated in a graded series of alcohol. Staining was then performed using the DAB chromogenic agent (Dako Corp, Carpinteria, CA). Negative control experiments were routinely performed.

The slides were scored independently by two experienced pathologists who were unaware of the origin of the slides. The semiquantitative scoring system suggested by Remmele and Stegner (9)considering staining intensity and percentage of positive cell nuclei was used for analysis of the immunohistochemical staining results. The staining intensity was described by scores between 0 and 3 (0 = no reaction, 1 = low, 2 = moderate, 3 = strong). Accordingly, the number of positive cell nuclei was counted and scored between 0 and 4 (0 = no positive cell nuclei, 1 ≤ 25% positive cell nuclei, 2 = 26–50% positive cell nuclei, 3 = 51–75% positive cell nuclei, 4 ≥ 75% positive cell nuclei). The product of staining intensity and percentage of positive cell nuclei resulted in an overall score (IRS) between 0 and 12. Each sample was categorized by this rating score in which an overall score of 0–1 was taken to be negative (−), 2–3 as weak (+), 4–6 as moderate (++), and >6 as strong (+++). We defined score >6 as high score and 0–6 as low score.

### Cell Culture

BPH-1, LNCaP, and PC3 cells were purchased from the ATCC (American Type Culture Collection, Manassas, VA). Cells were cultured in RPMI 1640 medium supplemented with 10% fetal bovine serum (GIBCO, MA, USA) at 37°C with 5% CO_2_. Cells were treated with 1 μM GSK-2879552 (MedChemExpress, China) or SP-2509 (MedChemExpress, China) for 72 h.

### Plasmid Construction and Cell Transfection

Plasmid was extracted using a Small Plasmid Extraction Kit (EM101, TransGen Biotech, Beijing) after vaccination and amplification of single colonies and bacterial fluid was sequencing verified. 293T cells were co-transfected with pLVX-FBXW7-ZsGreen-Puro (recombinant plasmid) to obtain high titer lentiviral (rLV-FBXW7) containing target gene using a Lentiviral packaging kit (R003, Wuhan Viraltherapy Technologies Co. Ltd). When cells reached 80–90% confluence, a total of 5×10^5^ cells/ml cells were transferred to cell plates, which were transfected by rLV-FBXW7 on the basis of MOI=20 the next day. Two days after transfection, lentivirus-infected cells were selected using complete medium (DMEM supplemented with 10% fetal bovine serum and 1% penicillin-streptomycin) containing 10μg/ml of puromycin.

siRNA was constructed and obtained from Wuhan Viraltherapy Technologies Co. Ltd. siRNA (100nM) was transfected into cells using Lipofectamine RNA iMAX (Invitrogen) for at least 48h; cells were then harvested for RNA and protein preparation.

### Cell Viability

Cell viability was assessed using a CCK-8 assay (Beyotime Biotechnology, #C0037) according to the manufacturer’s instructions. Briefly, cells were seeded into 96-well plates at a density of 5 × 10^3^ cells/well in 96-well plates. After overnight culture, the cells were transfected with plasmid vector or vehicle to overexpress FBXW7. After this process, cells were incubated with CCK-8 reagent. Then, cell viability was evaluated by absorbance measurements using a microplate reader (Molecular Devices, USA) at 450 nm.

### Western Blot Analyses

The protein expression levels of LSD1, FBXW7, c-MYC, NOTCH-1, and GADPH were examined by Western blotting. Briefly, proteins were separated on 8% SDS-PAGE gels (50 μg/lane) and then transferred to nitrocellulose membrane (Bio-Rad, Hercules, CA). The membranes were blocked with 5% non-fat milk in TBST buffer (10 mmol/L Tris–HCl, i0.15 mol/L NaCl, and 0.05% Tween 20, pH 7.2) for 2 h and incubated with primary antibodies overnight at 4°C. Primary antibodies used here were polyclonal rabbit antibodies against LSD1 (1:1,000 dilution; Affinity), FBXW7 (1:1,000 dilution; Affinity), c-MYC (1:1,000 dilution; Affinity), NOTCH-1 (1:1,000 dilution; Affinity), and GADPH (1:1,000 dilution; Affinity). After extensive washing with TBST buffer, the membranes were incubated with HRP-conjugated anti-rabbit secondary antibodies (1:50,000 dilution; Boster Biological Technology Co. Ltd). The proteins were detected using an enhanced chemiluminescence system (ECL Kit, Pierce Biotechnology, Beijing, China) and captured on light-sensitive X-ray film (Carestream, Xiamen, China). Optical densities were detected using ImageJ software.

### Quantitative Real-Time PCR

Total RNA was isolated from cancer cell lines using TRIzol reagent (Invitrogen, USA). The purity of RNA was examined by spectrophotometry and the first strand cDNA was synthesized using reverse transcription Reagents (ABI, CA) or the TaqManH MicroRNA Reverse Transcription Kit (ABI, CA) following the manufacturer’s instructions. QRT-PCR was performed using SYBRH Select Master Mix for CFX (Invitrogen) and using the CFX Connet TM real-time PCR system (Bio-Rad, USA). All results were normalized to the expression of GAPDH or snRNA U6. The quantitative analysis was calculated by using 2^−ΔΔCt^ method. All the primers are shown in **Table 1**.

**Table 1:** Indicated primers used in PCR experiments.

GENE	Primer sequences (5′-3′)
FBXW7	F: AGAGGAGGAACAGCAACAGC R: TGGGGAGGAGAGTTGGTGAA
GAPDH	F: TGAAGGTCGGTGTGAACGGATTTGGTC R: CATGTAGGCCATGAGGTCCACCAC

### Statistical Analysis

All data are expressed as the mean ± SEM. Statistical analyses involved one-way ANOVA and Tukey’s multiple comparisons tests. P<0.05 was considered to indicate a statistically significant difference.

## Results

### LSD1 Expression Is Up-Regulation While FBXW7 Expression Is Down-Regulation in PCa

**Figure 1:**
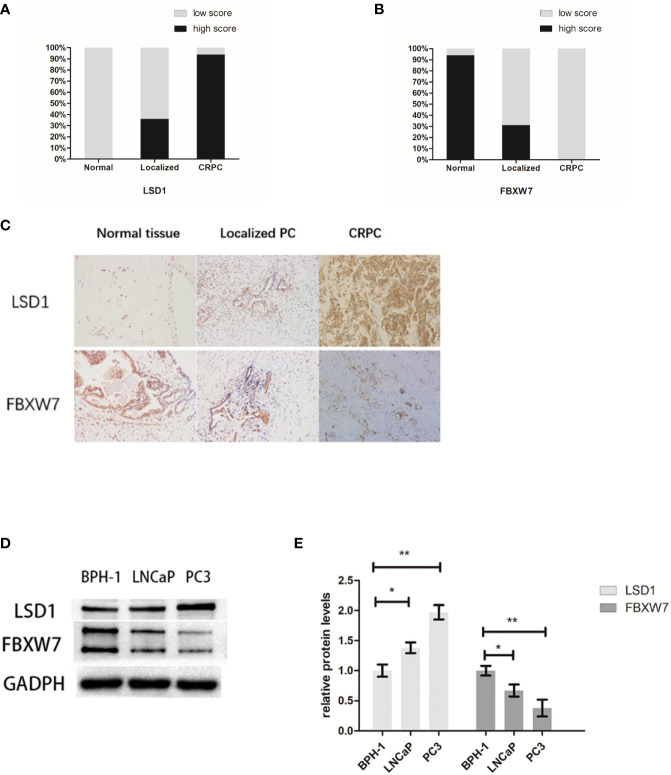
LSD1 expression is up-regulation while FBXW7 expression is down-regulation in PCa. **(A, B)** Proportions of high intensity scores of LSD1 and FBXW7 IHC staining in normal prostate tissues (n=52), localized PCa (n=36), and CRPCa (n = 16) samples are shown. **(C)** Representative IHC images of expression levels of LSD1 and FBXW7 in three different tissues. **(D)** Representative Western blot analysis of LSD1 and FBXW7 in indicated cell lines. **(E)** Relative protein levels of LSD1 and FBXW7 (compared with BPH-1 cells). Values are expressed as the mean ± SEM.*P < 0.05, **P<0.01, normalized by GADPH, relative to the BPH-1 group, n = 3.

In previous reports, LSD1 is highly expressed in various type of prostate cancer (10, 11) while the expression of FBXW7 decreased in prostate cancer (12). In order to explore the relationship between them, we used immunohistochemistry (IHC) to quantitatively analyze the expression of LSD1 and FBXW7 in normal tissues and primary and metastatic CRPC tumors. Immunohistochemical staining showed that LSD1 and FBXW7 proteins are both mainly localized in nuclei of luminal cells of prostate carcinoma cells. In 16 CRPC cases, LSD1 protein was expressed strongly and diffusely, while the expression level of FBXW7 is opposite (**Figure 1C**). The expression of LSD1 in CRPC tissues was significantly up-regulated compared with HSPC and normal tissues (**Figure 1A**). However, the expression of FBXW7 in PCa was significantly down-regulated (**Figure 1B**). The expression of FBXW7 in CRPC was significantly lower than that of HSPC and normal prostate tissues. We used Spearman’s rank correlation test to analyze the relationship between LSD1 and FBXW7 expression levels based on the overall staining score. As shown in **Table 2**, FBXW7 expression in the PCa specimens was negatively correlated to LSD1 expression (rs = −0.704, P<0.01). Furthermore, we explored expression levels of FBXW7 and LSD1 in different prostate cell lines. In PCa cell lines (LNCaP and PC3), LSD1 level was higher and FBXW7 level was lower compared with BPH-1 cells (**Figures 1D, E**).

**Table 2:** Correlation analysis between LSD1 and FBXW7 immunoreactivity in PCa specimens.

LSD1 expression	FBXW7 expression					rs	P
	+++	++	+	–	Total	−0.704	<0.01
+++	1	6	19	2	28		
++	10	1	7	1	19		
+	47	4	4	0	55		
–	2	0	0	0	2		
Total	60	11	30	3	104		

An overall score of 0–1 was taken to be negative (−), 2–3 as weak (+), 4–6 as moderate (++), and >6 as strong (+++).

### FBXW7 Suppresses the Viability of PCa Cells

**Figure 2:**
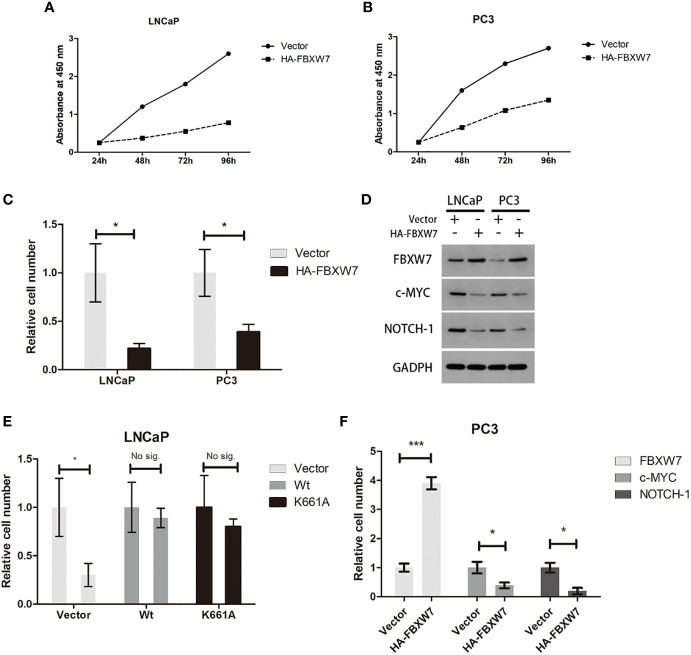
Increasing FBXW7 protein level suppress PCa cell viability by inhibiting key human cancer proteins. **(A**–**C)** The relative cell number of two cell lines after FBXW7 overexpression by plasmid vectors. **(D)** The result of immunoblotting performed by indicated antibodies. **(E**, **F)** The change of relative expression level of each protein compared by control group. Values are expressed as the mean ± SEM.*P < 0.05, ***P<0.001, n = 3, relative to the vector group. Protein levels were normalized by GADPH.

Previous study revealed that the expression of FBXW7 protein in prostate cancer was markedly reduced (12) compared with normal tissues. However, its effect on prostate cancer cells has not been confirmed. To further investigate the role of fbxw7 in the survival of prostate cancer cells, we overexpressed FBXW7 in different cell lines (LNCaP and PC3) and observed its effect on prostate cancer cell lines. We used plasmid vectors to overexpress FBXW7 in both cell lines. Then, the changes of FBXW7 protein levels were detected by immunoblotting. In each case, even in AR-null PC3 CRPC cells, FBXW7 overexpression decreased cell viability (**Figures 2A, B**). Compared with the control group, the protein level of FBXW7 increased and the cell count decreased after FBXW7 overexpression (**Figure 2C**). FBXW7 is considered as a typical tumor suppressor. It can target a series of key human cancer proteins, such as cyclin E, c-JUN, c-MYC, NOTCH-1, and MCL-1 (8). Then we detected some substrates protein expression of FBXW7. Comparing the control group and the FBXW7 overexpression group, FBXW7 overexpression decreased the expression level of its target proteins (c-MYC, NOTCH-1) (**Figures 2D–F**). It was suggested that FBXW7 may affect PCa cell viability by suppressing target ooncoproteins. Therefore, FBXW7 should be an independent factor that affects the survival of prostate cancer cells.

### LSD1 Promotes Prostate Cancer Cell Survival by Negatively Regulating FBXW7 Level

**Figure 3:**
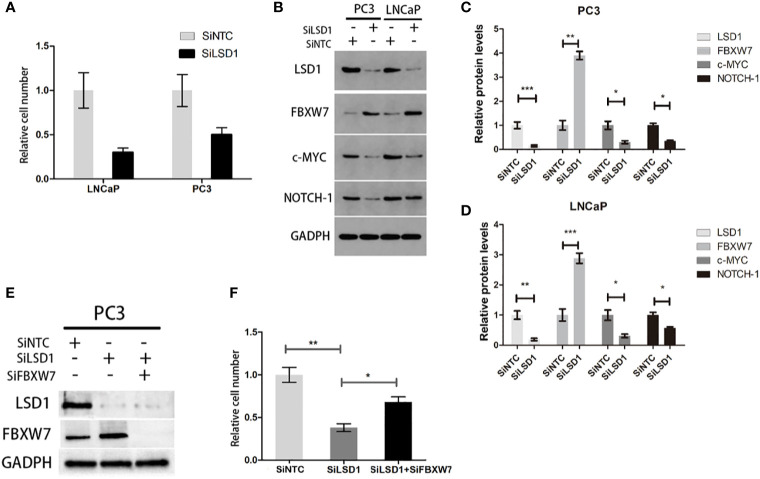
LSD1 promotes prostate cancer cell survival by destabilizing FBXW7 at post-translational level. **(A)** The relative cell number of LNCaP and PC3 cell lines was detected by cell counter after transfected with SiNTC or SiLSD1. **(B)** Representative bands of Western blot analysis for the expression of LSD1, FBXW7, c-MYC, and NOTCH-1 in indicated groups. **(C, D)** Relative expression level of each protein in **(B)** (SiNTC *vs*. SiLSD1). **(E)** Western Blot analysis for the protein expression of LSD1 and FBXW7 in rescue assay. **(F)** Relative cell number of indicated groups in rescue assay. Values are expressed as the mean ± SEM.*P < 0.05, **P<0.01, ***P<0.001, relative to the SiNTC group, n = 3. Protein levels were normalized by GADPH.

It is clear that the effect of LSD1 on histone demethylation in PCa. And previous studies have shown that LSD1 has some un-demethylated roles. To further examine the role of LSD1 in prostate cancer cell survival, we suppressed LSD1 with RNAi in different cell lines (LNCaP and PC3). The result showed that cell viabilities were significantly reduced in LNCaP and PC3 cells (**Figure 3A**). According to a recent report, LSD1 can negatively regulate the stability of FBXW7 protein independently of demethylation function (7). To further investigate the relationship between LSD1 and FBXW7 in PCa, we detected the level of LSD1 and FBXW7 after LSD1 knock-down. The data showed that LSD1 silence promoted the expression of FBXW7 on protein level and down-regulated the expression of down-stream target proteins (c-MYC, NOTCH-1) of FBXW7 in both cell lines (**Figures 3B–D**). In order to verify whether LSD1-FBXW7 interaction axis contributed to oncogenic effect of LSD1 in PCa, we performed a rescue assay (SiLSD1 *vs*. SiFBXW7+SiLSD1). Results suggested that viability partly recovered after combination with FBXW7 knockdown (**Figures 3E, F**). Taken together, these results demonstrated that LSD1 may negatively regulate FBXW7 for cancer cell survival in PCa cells.

### LSD1 Destabilizes FBXW7 Independent of Demethylation-Function in PCa Cells

**Figure 4:**
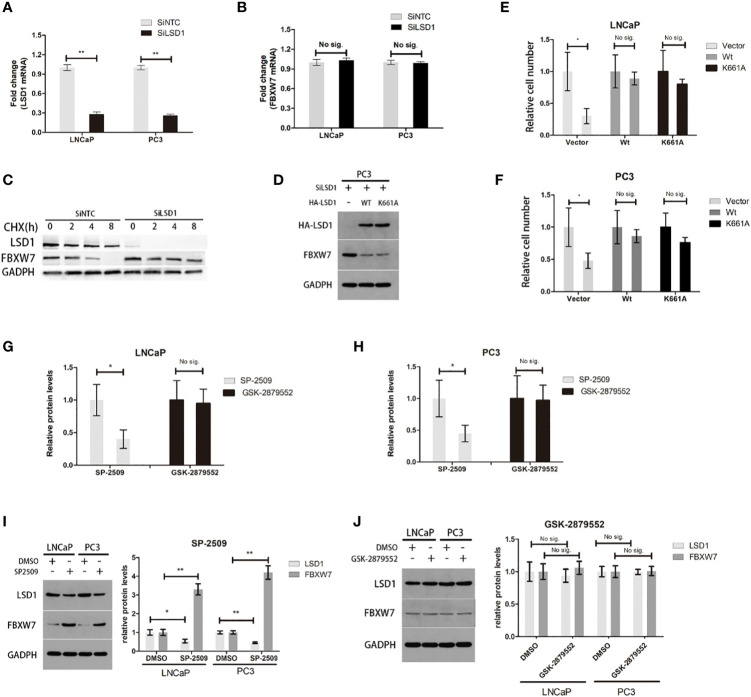
It is independent of demethylation-function that LSD1 can destabilize FBXW7 in PCa. **(A, B)** The change of LSD1 and FBXW7 mRNA levels were detected by qPCR after LSD1 knockdown. **(C)** LSD1 and FBXXW7 half-life were detected by western blotting analysis at various time points, such as 0, 2, 4, and 8h after treatment of CHX. **(D)** Western blot analysis was used to detect protein expression level of LSD1 and FBXW7 in PC3 cells that were transfected with indicated exogenous protein. **(E, F)** Relative cell number of LNCaP and PC3 cells after transfected with exogenous wild-type LSD1 or catalytically deficient mutant K661A. Wild-type and catalytically deficient mutant LSD1 both can decrease FBXW7 protein level after transfected with SiLSD1 in PC3 cells. **(G, H)** Relative cell number of both cell lines after treated with SP-2509 or GSK-2879552 for 72h. **(I, J)** The relative protein expression level of LSD1 and FBXW7 in both cell lines after the treatment of inhibitors. The simple catalytic inhibitor GSK-2879552 cause no significant change. And SP-2509 cause the LSD1 depletion which results in up-regulation of FBXW7 expression level. This result is absent in GSK-2879552 treated group. Values are expressed as the mean ± SEM.*P < 0.05, **P<0.01, relative to the vector or DMSO group, n = 3. Protein levels were normalized by GADPH.

To further investigate the underlying mechanism that LSD1 regulate the expression of FBXW7, we explored the interaction between above two. Previously, LSD1 was deemed as a hypothetical AR cofactor, which can regulate target proteins mRNA level through histone demethylation (13). Therefore, we used qPCR to detect the mRNA levels of LSD1 and FBXW7, and found that LSD1 silence did not significantly change the mRNA level of FBXW7 (**Figures 4A, B**), indicating that LSD1 inhibits the expression of FBXW7 protein at post-translational level. Then, we determined whether LSD1 affected the FBXW7 protein half-life. The result showed that depletion of LSD1 significantly extended the protein half-life of endogenous FBXW7 (**Figure 4C**). However, LSD1 can also demethylate non-histone substrates (14–16). To clarify whether LSD1’s demethylase function contributes to the change of FBXW7, we complemented cells with ectopic wild-type LSD1 or with K661A (catalytically deficient mutant LSD1 (17)) after LSD1 knockdown (**Figure 4D**). Overexpression of either can abrogate the effects of LSD1 RNAi on reducing cell survival or the expression of FBXW7 (**Figures 4E, F**), demonstrating that demethylation function of LSD1 is not indispensable for PCa. More importantly, we found that SP-2509 (Allosteric inhibitor) treatment blocked the LSD1–FBXW7 interaction, which is an effect that GSK-2879552 (catalytic inhibitor) cannot achieve. The result showed that SP-2509 recapitulated the effects of LSD1 RNAi on cell viabilities (**Figure 4G**). But the inhibitory effect of GSK on PCa cells is not satisfactory. The treatment of SP-2509 reduced LSD1 level and increased FBXW7 level in both cell lines (**Figure 4I**), while either did not have significant change after GSK-2879552 treatment (**Figure 4J**).

## Discussion

In the past, it was believed that LSD1 played a role in prostate cancer by regulating the transcriptional activity of AR, because LSD1 can remove the methylation of the transcriptional repressive marker H3K9me1/2 (18–20). However, Sehrawat et al. found that LSD1 can cooperate with ZNF217 to activate some gene networks in lethal prostate cancer. Most importantly, this effect does not depend on its demethylase activity and AR pathway (11). They demonstrated that LSD1 can promote the survival of a lethal prostate cancer cell model independently of the function of demethylase, and identified SP-2509, a drug that blocks the independent function of demethylase. Since then, more and more studies have reported that LSD1 exerts this atypical function and affects cancer cell processes. However, the role of these functions in prostate cancer has not been clearly elucidated.

In conclusion, we demonstrate a new role of LSD1 in prostate cancer in our study. First, we determined that LSD1 up-regulation is ubiquitous in lethal CRPC samples and FBXW7 level is down-regulated. Moreover, LSD1 inhibition resulted in an increase of FBXW7 protein levels, while FBXW7 mRNA levels did not increase. We demonstrate that LSD1 can promote the survival of prostate cancer cells by down-regulating FBXW7 protein levels. More importantly, the demethylase activity inhibitor GSK-2879552 cannot eliminate this effect between LSD1 and FBXW7, while the allosteric inhibitor SP-2509 is effective. These results indicate that demethylation function of LSD1 may be dispensable in CRPCa. However, the mechanism that SP-2509 reverse the effect of LSD1-FBXW7 interaction was not fully elucidated. It may be ascribed to the reduction of LSD1 or the alteration of LSD1 conformation due to the binding of inhibitor. These results showed that in the development of LSD1 targeted drugs, we should further look for inhibitors of demethylase-independent functions. However, these hypotheses were limited, as they were mainly based on a retrospective study. Better designed prospective studies and further experiments *in vitro* and *in vivo* assays are needed to confirm this research.

LSD1 is abnormally expressed in a variety of tumors and is often associated with poor prognosis, it is often considered as a potential anti-cancer treatment target. Accordingly, a line of LSD1 inhibitors have been in clinical studies, such as ORY-1001, RG6016, INCB059872, and so on. And most of these inhibitors are based on blocking its demethylase activity. However, recent studies showed that LSD1 is also involved in a series of protein-protein interactions that are independent of its demethylation function (21). The functional diversity of LSD1 is supported by its complex structure which enables it interact with many endogenous proteins. This role can also be involved in cancer development. Therefore, catalytic inhibitors of LSD1 are often difficult to suppress the survival of cell models sensitive to LSD1 RNAi (11). This is a newly discovered mechanism that LSD1 promotes tumorigenesis and development by protein-protein interaction. This discovery has greatly expanded the scope of LSD1 biological functions. Compared with typical functions of LSD1, there are few researches on its such functions at present. As mentioned above, as more functions of LSD1 are gradually discovered, the effort of drug research should not be limited in inhibiting its demethylase activity. Research on inhibitors that are more potent, more specific and can block the atypical functions of LSD1 will become a new direction for the design of LSD1 targeted drugs in the future.

## Data Availability Statement

The original contributions presented in the study are included in the article/supplementary material. Further inquiries can be directed to the corresponding author.

## Ethics Statement

The studies involving human participants were reviewed and approved by Renmin hospital of Wuhan University Ethics Committee. The patients/participants provided their written informed consent to participate in this study.

## Author Contributions

X-kQ designed the study and experimental studies. YD edited and prepared the manuscript. X-hL was the guarantor of integrity of the entire study. LW performed the statistical analysis. All authors contributed to the article and approved the submitted version.

## Funding

This paper is funded by the National Natural Science Foundation of China (No. 81972408 and No. 82000639), the frontier project of Wuhan Applied Foundation (No. 2018060401011321), and Innovation Project of Medical School of Wuhan University (TFZZ2018017).

## Conflict of Interest

The authors declare that the research was conducted in the absence of any commercial or financial relationships that could be construed as a potential conflict of interest.
